# Post-healing perceptions and experiences of alcohol withdrawal and life management in men with alcoholic pancreatitis: a qualitative study

**DOI:** 10.3389/fpsyg.2023.1192835

**Published:** 2023-08-16

**Authors:** Runpeng Chen, Qinghua Wang, Dongyang Wang, Xinyue Liu, Haiyun Wang, Jiaoyang Guo, Xinghui Liu

**Affiliations:** ^1^Department of Nursing, Binzhou Medical University, Yantai, Shandong Province, China; ^2^Faculty of Nursing, Mahidol University, Nakhon Pathom, Thailand; ^3^Department of Nursing, The Third People’s Provincial Hospital of Henan Province, Zhengzhou, Henan Province, China; ^4^Shengli Oilfield Central Hospital, Dongying, Shandong Province, China; ^5^Shandong Vheng Data Technology Co., Ltd., Yantai, Shandong Province, China

**Keywords:** alcoholic pancreatitis, alcohol withdrawal, life management, cognition, experience

## Abstract

**Introduction:**

The aim of this study was to examine the perceptions and experiences of male patients with alcoholic pancreatitis after healing regarding alcohol withdrawal and life management.

**Methods:**

This study used a qualitative descriptive design, and participants were selected by purposive sampling from two tertiary care hospitals in Shandong Province, China. Semi-structured in-depth interviews were conducted with 18 male patients discharged from the gastroenterology department who had recovered from alcoholic pancreatitis. Colaizzi’s method was used to analyze the interview data, and the findings were reported using COREQ criteria.

**Results:**

By analyzing the interview data, we summarized five themes, (1) the dilemma of sobriety, (2) role change, (3) illness status, (4) family influence, and (5) life management.

**Conclusion:**

By profiling the perceptions and experiences of post-healing alcoholic pancreatitis patients’ alcohol cessation and life management in men, it helps to grasp the details of alcohol cessation and health direction of patients’ home management, which provides more directional guidance to help patients maintain positive and good lifestyle habits and active management awareness, followed by targeted personalized interventions to provide patients with knowledge of disease care and health management.

## 1. Introduction

Alcoholic pancreatitis (AP) is caused by an excessive dependence on psychoactive substances containing alcohol, most commonly alcohol abuse. Men have the X-linked CLDN2 gene variant of the AP allele risk, which increases their susceptibility to acute alcoholic pancreatitis (Nikkola et al., 2022). Currently, there are no clear and uniform diagnostic criteria for alcoholic acute pancreatitis, whereas men with alcoholic chronic pancreatitis (ACP) are usually described as having a high alcohol intake of >80 g/d for 2 or more years in the absence of other factors (Wang et al., 2023), which predisposes them to ACP development.

Only 3 to 5% of heavy drinkers develop alcoholic acute pancreatitis (Nikkola et al., 2022). However, ACP is more common in eastern China (*r* = 0.04, *p* = 0.006), with a chronic pancreatitis incidence of 18.8% (Wang et al., 2021). The substantial clinical manifestations are abdominal pain, pancreatic stones, diabetes mellitus, and diarrhea; the symptoms are usually more severe than those of non-alcoholic pancreatitis (Wang et al., 2023). In North America, AP has a high incidence of approximately 45% (Conwell et al., 2017). AP prevalence is also greater in Europe, especially in Eastern Europe (Roberts et al., 2017). Studies have revealed that 80% of perennial alcoholics have AP (Hayduchok et al., 2022), with a global incidence of 9.62/100,000 and a mortality rate of 0.09/100,000 (Xiao et al., 2016). Moreover, the disease risk is higher with liquor consumption than with beer or wine consumption (Gapstur et al., 2011); alcohol and smoking increase the disease risk (Muniraj et al., 2014). One study revealed that AP incidence increased from 0.72 to 5.19% between 1961 and 2016 (Iannuzzi et al., 2022). Some studies have revealed that people’s lives have gradually become more stressful in recent years, and alcohol consumption increases yearly. Further, there has been an increase in alcohol consumption among women, which has led to an increased morbidity and mortality rate of AP, burdening society and the health care system (Brezovec et al., 2022; Chaudhry et al., 2023).

Persistent abdominal pain, malnutrition, complications, difficulties in quitting alcohol, and financial burden are all important factors that contribute to the reduced quality of life of patients with AP (Ji, 2012; Singh et al., 2022) and seriously affect the patients’ organic condition, psychological health, family relationships, and social roles. Some studies claim that abstinence from alcohol can greatly reduce the risk of AP progression (Beyer et al., 2020). Patients with healed AP are required to abstain from alcohol and undergo self-management at home. If patients do not abstain from alcohol, or if interventions and management are unreasonable and untimely, it will lead to long-term disease migration without healing, with a high risk of recurrence and progression to pancreatic cancer, which seriously affects patients’ physical and mental health. Therefore, a quality life management program is necessary to help patients quit drinking, improve their quality of life, avoid disease recurrence, and maintain emotional stability, psychological health, and family harmony (Lewis et al., 2018).

A study examined the perceived experience of patients with recurrent triglyceridemic AP regarding life management (Chen et al., 2022), which provides a reference for the development of follow-up interventions and lifestyle improvement aspects for such patients. Qualitative studies have also been conducted on the psychological experiences of patients hospitalized with AP, focusing on the description of the thoughts, feelings, and behaviors of hospitalized patients (Ma et al., 2022). Alcohol not only causes inflammation of the pancreas, but is also a risk factor for pancreatic cancer, and a retrospective cohort study that tested the age of diagnosis of pancreatic cancer in patients exposed to specific risk factors found that there was a significant correlation between the age of initiation of alcohol consumption and the age of diagnosis of having pancreatic cancer (Lin et al., 2022), suggesting that abstinence from alcohol is essential for the prevention of disease recurrence and cancer. For patients with AP, abstinence and life management are long processes. After experiencing the first or multiple episodes, hospitalization, and home management, patients have their unique disease perceptions and life experiences. Their feelings about abstinence and life management vary, the details of which are noteworthy. Most previous qualitative studies have focused on the symptoms, psychology, life experiences, life dilemmas, and influencing factors of patients with acute and chronic pancreatitis (Fitzsimmons et al., 2005; Cronin and Begley, 2013; Liu and Zhang, 2020); however, few studies have addressed the perceptions and experiences of patients with AP with alcohol withdrawal and home life management in men. The purpose of this study was to explore the perceptions and experiences of alcohol abstinence and home life management in male patients managed at home after healing from alcoholic pancreatitis, and to conduct a qualitative descriptive analysis.

## 2. Methods

### 2.1. Procedure

We used a face-to-face interview format and selected 18 patients with AP who were discharged from two tertiary care hospitals in Shandong Province after December 2022 for rehabilitation, of whom 10 patients’ family members also participated with the patients; all interviews were conducted separately. The included patients were all men: aged 18 years or older; could communicate in Mandarin; met the diagnostic criteria for ACP (2 years or more and alcohol intake >80 g/d); and had an AUDIT-C (Alcohol Use Disorders Identification Test) score ≥ 3. We excluded patients with cognitive impairment, hearing impairment, psychiatric disorders, and those with serious diseases, such as cancer or severe cardiopulmonary or liver disease.

### 2.2. Design

We used a descriptive qualitative research design with a semi-structured approach to interviewing based on purposive sampling, which facilitated the asking of new exploratory questions at the right time when new themes emerged during the interview and increased the detail and coherence of the interview. The research team members were registered nurses who had participated in training related to qualitative research and were certified to read all patients’ case histories in detail to grasp each patient’s condition before the interviews. We interviewed patients in five aspects: patients’ illness experience, drinking experience, social life, family influence, and psychological state. Three patients were pre-interviewed first, and then the interview outline was appropriately modified according to the specific interview format and content, with unlimited interview time until data saturation. The finalized interview outline is presented in Table 1. All patients signed the interview informed consent form and followed the Consolidated Criteria for Reporting Qualitative Research (Shenton, 2004; Tong et al., 2007) and credibility criteria procedures (Shenton, 2004). (1) Credibility: the researcher analyzed the semi-structured interviews with the researcher’s field notes and audio files, followed by a team meeting to compare analyses and identify themes, which later required participants to validate the data obtained during the data collection phase. (2) Transferability: an in-depth description of the study, providing detailed information and data on the researcher, participants, context, sampling strategy, and characteristics of the data collection and analysis procedures for comparison. (3) Reliability: reviewed by external researchers; the study protocol was evaluated and reviewed, focusing on the study methodology and design. (4) Confirmability: the use of triangulation to reduce the impact of researcher bias, self-reflection and critique for the purposes of the study, and timely recognition of flaws and potential impacts of the research methodology. Provide a detailed description of the methodology, explain the resulting data and structure, take a data-oriented approach, present the final data collected and processed with recommendations, and ensure the integrity of the findings.

**Table 1:** Theme and subtopic.

Theme	Subtopic			
The dilemma of sobriety	The torture of alcohol addiction	Risk–return trade-off	Stigmatization	Self-paralysis and stage control
Role change	Professional role struggles and entanglements	Lack of motivation	Patient Role Absence	
Illness status	Lack of disease knowledge	Non-compliance	Concerns about the risk of disease	
Family influence	Family’s helplessness and lack of care	Overprotection and overdependence		
Life management	Dilemma	Shift in social style	Adaptation	

### 2.3. Participants

The patients were all men with a mean age of 43.11 years (standard deviation = 9.791 years, range = 30–62 years). The 10 family members included the patients’ wives, children. Considering that the purpose of this study was to explore the experience and perception of alcohol cessation and life management in men with AP, no demographic information was collected for the family members. We collected patients’ demographic characteristics and baseline data, including age, sex, occupation, education, marital status, monthly income, and children. Twenty-two semi-structured interviews were conducted from January 17 to February 18, 2023, and family members were encouraged to participate in the interviews to reduce patient tension and increase their involvement in managing patients’ lives. Informed consent was obtained from patients and families for the interviews and audio recordings, and the interviews were transcribed verbatim within 24 h after each interview. The detailed demographic characteristics of all participants are presented in Table 2.

**Table 2:** Demographic characteristics (*n* = 18).

	*n* (%)
Age	
<40	8 (44.4%)
41 ~ 50	5 (27.8%)
51 ~ 60	4 (22.2%)
>60	1 (5.6%)
Sex	
Men	18 (100%)
Women	0 (0)
Career	
Farmers	3 (16.7%)
Staff	5 (27.8%)
Self-employed	3 (16.7%)
Administrators	3 (16.7%)
Businessmen	4 (22.2%)
Education level	
Elementary school and below	2 (11.1%)
Junior high school	1 (5.6%)
High school	4 (22.2%)
Specialist and above	11 (61.1%)
Marital status	
Married	14 (77.8%)
Unmarried	2 (11.1%)
Divorce	1 (5.6%)
bereaved spouse	1 (5.6%)
Monthly income	
<999	3 (16.7%)
1,000 ~ 7,999	3 (16.7%)
8,000 ~ 9,999	3 (16.7%)
10,000 ~ 19,999	5 (27.8%)
>20,000	4 (22.2%)
Children	
There are	14 (77.8%)
None	4 (22.2%)

### 2.4. Data analysis

We used the Colaizzi qualitative analysis procedure in this study (Vignato et al., 2022). The interview data were analyzed according to the previously reported steps (Lindgren et al., 2020) in Table 1.

### 2.5. Ethics approval and consent to participate

This study followed the Declaration of Helsinki (Shrestha and Dunn, 2020) (and was approved by the Affiliated Hospital of Binzhou Medical College and Shengli Oilfield Central Hospital, ethics number: 2). All participants were informed of the purpose of the study, and all patients participated voluntarily.

## 3. Results

From the interview data analysis, we summarized five themes, (1) the dilemma of sobriety, (2) role change, (3) illness status, (4) family influence, and (5) life management (Table 3).

**Table 3:** Colaizzi qualitative analysis steps.

Colaizzi qualitative analysis steps
An initial code was assigned to each patient, and each patient’s interview notes were read repeatedly in depth to capture their sobriety experience and the content of their life management plan.
To enhance the overall understanding of the dialogue, we refined, identified, and extracted the important statements from each patient’s interview and developed the meaning of all important statements.
Subsequently, the meaning of all important statements in the interviews was integrated and summarized into themes, subthemes, and detailed detail data sets through discussion and analysis by the research team members.
Interviews were conducted until the information was saturated and stopped when no new subject lines emerged.
The basic structure of the content of the interviewed patients’ perceptions and experiences of alcohol cessation and life management was discussed.
The parsed text data results were sent to the patient for validation.

### 3.1. Theme 1: the dilemma of sobriety

#### 3.1.1. The torture of alcohol addiction

Patients who had alcohol addiction before the disease were suddenly asked to stop drinking due to illness and physical reasons. They could not attend their usual drinking parties and gatherings and felt idle. The alcohol craving and inability to drink made them suffer and find it difficult to bear the situation and even doubt the value of their lives.

“I’ve been drinking for 20 years, and when I suddenly quit drinking, I felt simply unprepared, did not know what to do with my free time, and felt so uninspired to live” (N1).

“I cannot stand it, I cannot drink, I’m almost jumping up and down at home, I feel like I do not even know myself anymore!” (N6).

#### 3.1.2. Risk-return trade-off

In China, the hospitality of meeting friends with alcohol has a long history of being a lubricant for human interaction. Advocates of alcohol believe that the amount of alcohol consumed reflects how much you respect others and how much they value you. The greater the amount of alcohol consumed, the greater the engagement, sense of being needed, pride, self-satisfaction, and value. Therefore, alcohol is often linked to good relationships and workplace atmosphere, making people feel needed and valued (Lee et al., 2022). The trade-off for friendship, better social integration, a smoother career, wealth growth, and access to promotion is that some patients do not refuse alcohol, and the risk of disease recurrence is hidden under the socialization of alcoholism.

“China has drinking etiquette, and if you want to quit drinking, it can be difficult. As part of my job to attract investment, I often meet with clients. Sometimes they invite me to drink, but what if I choose not to? Would the customers feel that I’m not being respectful?” (N1).

“I want to control it properly, although it is said to have the intention to quit drinking, but it is likely to encounter again to push off the drinking scene, and we make friends basically first drink, now suddenly quit drinking, which is equal to get out of the circle, slowly are not taking you to play” (N2).

#### 3.1.3. Stigmatization

Some patients accidentally learned that they had been named “alcoholics” or “addicts” after their illness, which hurt their self-esteem. They felt discriminated against and humiliated and felt a lot of pressure and psychological burden, disappointment, discomfort, and frustration.

“My friends call me an addict and it makes me feel ashamed, aggrieved, I do not feel the same as everyone else and I’m in pain every day!” (N11).

#### 3.1.4. Self-paralysis and stage control

Some patients were uncertain about the outcome, even after life management. They considered living in the present rather than dwelling on uncertainties. However, they were sometimes eager to recover from their illness and implemented a regular lifestyle in stages. Possibly, they could not predict their next action.

“Is it that I cannot get well in the future, I cannot get well even if I am happy for 1 day, I think I want to drink enough before I die” (N9).

“Will I be able to live well in the future, I sometimes want to go to a regular life properly, but will that work? Will I still be able to live my life properly?” (N11).

### 3.2. Theme 2: role change

#### 3.2.1. Professional role struggles and entanglements

Some patients stated they needed to recuperate at home and could not participate in work due to their health. They remained skeptical and distrustful of their subordinates’ progress and ability to handle important issues. They were uncertain about their subordinates’ capabilities but could not intervene due to their illness. Thus had a sense of anxiety and frustration.

As employees, they were unable to complete their work tasks in time because of illness; they felt that their personal work value could not be realized, and they were worried that their careers would be affected, which increased their uncertainty and anxiety about their future career development.

“Since being hospitalized, my business is basically left to the people below me, and I am very uneasy and anxious, and I feel very annoyed inside” (N4).

“Originally said to go to work at the beginning of the year, the leadership has not seen me so far, delayed work, now layoffs are very strong, really afraid of being fired” (N6).

#### 3.2.2. Lack of motivation

Patients had to give more time and energy in the workplace to keep their families financially stable. They realized the importance of sobriety and life management. However, work demands diminished their patience and determination, making change difficult. Therefore, they had to sacrifice their health to make ends meet.

“I cannot bring myself to manage myself, I have a mortgage and a car payment to pay, and I have to work or there is no way to support my family” (N7).

#### 3.2.3. Patient role absence

After the illness, patients did not value their status as “patients” because their quality of life and status quo were not greatly affected, and they felt they only needed rest. Even if they suffered for a short period because of hospitalization, they recovered from the experience and did not feel a need to undergo life management, and decided to retain the status quo. Many patients even chose to numb themselves with alcohol due to the stress of their work life.

“There’s no thought of quitting drinking or exercising, I do not feel the need to because I need to work Monday through Friday and I run a couple of supermarkets and engineering firms, and you are asking me to exercise in my free time? I do not think I can do that, it’s good to keep it as it is” (N1).

“I originally also ate breakfast, now I do not eat at all, I eat irregularly, love to drink, love to eat late night snacks, and I am particularly good at cooking, want to eat at 10 pm I also go to do” (N17).

“I’m so tired, drinking relaxes me the most, and I do not know anything when I’m drunk” (N1).

### 3.3. Theme 3: illness status

#### 3.3.1. Lack of disease knowledge

Some patients observed that their friends who share their drinking habits did not develop the disease. Thus, they believed that their illness was related to other factors, such as diet and not alcohol consumption, and that the relationship, if any, was minimal. Adequate knowledge of the disease and health education may change their minds, depending on the patient’s correction of the disease and self-perception; however, the process may be slow.

“I’ve been drinking for 18 years, and although I drink a lot each time, I only drink intermittently, sometimes not more than a few times a month, and that’s what I do with my friends, so the illness is definitely not due to drinking” (N12).

#### 3.3.2. Non-compliance

Many patients, knowing that they could not drink alcohol, still did not refuse alcohol at compulsory drinking parties. They intended to attend the party without drinking but easily resumed drinking, neglecting the disease risk.

“I thought there should be no problem ah, I feel quite well nourished this time, the last attack was 7 months ago, last time I drank five glasses of white wine, this time I just drank two glasses, I did not expect to have another offense” (N6).

#### 3.3.3. Concerns about the risk of disease

Some patients were discharged from the hospital in poor physical condition, losing weight daily. Their experience of repeated admissions and ICU (Intensive Care Unit) stays due to recurrent pancreatitis caused by alcohol consumption made them have palpitations. They lived carefully, controlling their symptoms with medications to maintain their basic living condition. They felt that pancreatic cancer could separate them from their families.

“After a long time of recuperating at home and feeling better, I accidentally drank some cold water, then the pain and swelling started again, but it came and went quickly, and it got better again after resting for a while. What should I do, can I get well, and will I get sick again?” (N3).

“My pancreatic function is much damaged and I feel very uncomfortable every day. Usually I have to use insulin to maintain my blood sugar and the doctor also told me to supplement pancreatic enzymes, which are good for me to control my disease, but can they fight cancer? My daughter just finished her college entrance exams this year, can I last until she graduates from college?”(N7).

### 3.4. Theme 4: family influence

#### 3.4.1. Family’s helplessness and lack of care

Some patients’ families expressed their views that their sick family members were dismissive of sobriety and life management and ignorant of the condition. They stated that they could not make any decisions or efforts in their families’ place, initially with anger, which slowly became profound helplessness. Sometimes they were too busy taking care of their children and dealing with other family matters to take care of their patients, who had to take care of themselves most of the time.

“I know what’s at stake, but he does not listen, and I cannot keep an eye on him, and I’m afraid he’ll still do what he wants when he gets home (sigh)” (F7).

“They can not help me ah, we have two children, my love has to take care of the children, I have to cook for myself, and sometimes I am very busy, can not return home, how to manage life? I do not know (said with a bitter smile)” (N2).

#### 3.4.2. Overprotection and overdependence

Individual patients’ families described how the patient was young and immature. They believed it was a bit overwhelming to ask someone who was not yet capable of disciplining himself properly to accomplish the tasks of sobriety and health management, and that he needed to be well protected and needed more family help to accomplish these onerous management tasks when in reality, the so-called “little friend “was 42 years old.

Some patients believed that they were incapable of managing their lives and needed to rely on their family (parents). They believed that the key to achieving sobriety was discipline from family members. They were wrongly focused; possibly, they needed the right knowledge of self first.

“He’s still young and I do not think he can do it on his own” (N4).

“My mom wants to come over to my house to spy on me and keep me from drinking, and I actually feel the need for my mom to come over” (N10).

### 3.5. Theme 5: life management

#### 3.5.1. Dilemma

Many patients stated that they were physically and mentally exhausted; the physical fatigue was due to life activities, and the psychological aspect was also substantial. They stated that resisting the temptation of alcohol, tobacco, and food was exhausting them and caused great distress; the effect of life management was not good, and their physical state did not improve, which made them feel caught in a foggy life dilemma and at a loss.

“I cannot hold on anymore, it’s not easy for me to maintain such a state of life, I feel so tired of living, I really do not know what to do next!” (N15).

#### 3.5.2. Shift in social style

Some patients took advantage of their illness to put off all meaningless drinking parties and took the initiative to change their social style, for example, from drinking alcohol to tea and changing the venue of certain business meetings to a sports field, which produced good social results. Thus, they were better adapted to life, in a positive state, and more flexible in the world. This was mostly observed in younger patients, who were generally more educated and with better cognitive capabilities, confident in a good life and healthy body, and hopeful for the future; thus, they could make positive changes.

“From now on, just take this disease and put off the drinking game, try not to drink any more, just drink tea” (N11).

“I was very aware of my condition, I had to stop drinking for myself, and I changed a lot of business meeting places, and the other person was able to understand and accept it” (N3).

#### 3.5.3. Adaptation

Some patients had the courage to change, the determination to overcome difficulties, and the fearlessness to sacrifice. They reconciled with themselves, found a balance between life and management after moving away from the “paper life,” stabilized their illness, rediscovered the meaning of life, created another career, and rediscovered their values. They increased their companionship and mutual understanding with their families. They were increasingly involved in family affairs and children’s education, resulting in increasingly harmonious family relationships, greatly increasing their happiness and satisfaction.

“I have confidence in myself, I have succeeded in quitting drinking, my family is very attached to me, and this admission has given me a wake up call to take more care of myself in the future” (N4).

“My lover says I’ve been much more diligent lately and do a lot of housework well, before I did not like to do housework” (N11).

## 4. Discussion

In this study, a subset of patients who made attempts to quit alcohol experienced significant alcohol withdrawal symptoms and struggled to overcome their addiction. Unfortunately, their efforts to quit were hampered by a lack of willpower. Alcoholism led to the development of various severe health conditions such as pancreatic disease, liver disease, cancer, brain damage, anxiety, and depression, among others. Despite the potential life-threatening consequences, a majority of these patients still did not choose to stop drinking. In addition to a weakened willpower, it remains unclear why men in particular find it challenging to quit alcohol consumption. Studies state that alcohol often plays an important role in male-dominated religions or rituals. Drinking is also an important channel for men to achieve social cohesion and involvement in the workplace and is pivotal in men’s social and political careers (Nwosu et al., 2022). In addition, in China, there is a deep-rooted drinking culture, a traditional habit that has been passed down for millennia and is driven by a traditional value of respect for teachers and hospitality and friendliness, which has slowly evolved from what was originally a polite act of kindness into a social requirement (Lee et al., 2022). These sociocultural factors play a significant role in patients’ decision-making process, as they carefully evaluate the various influences, benefits, and potential risks associated with abstaining from alcohol in light of their existential realities. Unfortunately, a large majority of patients find it challenging to withstand the pressures and temptations of everyday life, making it extremely difficult for them to refuse alcohol. Consequently, this difficulty often leads to unsuccessful attempts at quitting alcohol.

Some patients in this study were called “alcoholics” and “addicts” by others. The stigmatization of people with substance use disorders is a common phenomenon today, and some mass media even label people with alcohol problems as such. Studies have revealed that patients affected by alcohol use problems are perceived as having more negative effects than patients with other medical-like disorders (Broyles et al., 2014); this perspective can result in some punitive public judgments against people with drinking problems that can view alcoholics as intentionally behaving inappropriately, resulting in inappropriate language is expressed against them and subsequently spreading socialized stigmas that undermine patients’ dignity and identity (Zwick et al., 2020). This stigma can be stressful and have a substantial negative psychological impact on patients. Sandra et al. conducted a quantitative study and reported that stigmatization could harm patients’ self-esteem and diminish their quality of life (Oliveira et al., 2016). Some studies have even interpreted “addiction” as a character flaw or weakness (Nieweglowski et al., 2018). Other studies have revealed that stigma is often strongly associated with terms such as alcohol and substance abuse, which increases depression, shame, and self-doubt, and undermines patients’ confidence and self-esteem, preventing them from making accurate judgments about themselves (Volkow et al., 2021), has a negative effect on health status and is strongly associated with reduced quality of life (Carol et al., 2022), which is consistent with our findings. Some of the patients in this study felt negative comments from the society, after friends or family members used stigmatizing words to them by chance, maybe they did not mean any harm, but the patients thought that their family members and friends were stigmatizing and caricaturing them, so they neglected their family’s love and care, and doubted their friendship with their friends, and thought that they did not really care about them. They feel that they are different from other people, and the inferiority complex brought about by this difference confuses them, generating self-paralysis and avoidance behaviors, and even self-abandonment, leading to a lack of strong will to quit, and in the face of the difficulty in quitting and lack of clarity about the direction of their condition, they are easily anxious or depressed, which seriously affects their quality of life.

Some of the patients in this study were caught up in varying degrees of occupational role struggles in the early stages of healing. They remain attached to the work progress of employees, or they are in a dilemma because they cannot finish their work in time. They feel that their degree of accomplishment of goals and sense of conviction is greatly reduced; this situation is described as reduced self-efficacy and is the main reason they lack the motivation to change their lives and fall into a professional role struggle, eventually leading to role change failure. Self-efficacy is centered on personal agency, is triggered by actions taken by the individual, and it both influences and is influenced by behavioral and environmental factors of the patient (Graham, 2022). When the patient believe he can stop drinking and manage his life but has no practice, he falls into a paradox of consciousness and behavior, leading to severe role struggles and a lack of motivation. Under the influence of these factors, even if the patient is motivated to change, he is limited. Some studies claim that occupational self-efficacy positively correlates with life satisfaction in China (Jiang et al., 2017). In this study, problems such as role ambiguity and conflict due to illness can trigger low self-efficacy in terms of dissatisfaction with work status. The occupational role struggle and lack of role behavior caused by this low self-efficacy can create a impediment for patients to engage in a disciplined alcohol cessation and management program. And even lead to severe mental health problems (Mérida-López et al., 2017). Moreover, the resignation that the social status and professional progress gained through one’s own efforts and with the assistance of alcohol may be slowly fading due to the condition may also account for the patient’s lack of motivation to abstain from alcohol, and the paradox of subjective initiative but lack of practice presents the patient with an opportunity to avoid change.

Some patients are unaware of pancreatitis and stubbornly judge the disease as unrelated to alcohol consumption based on their life experiences. The lack of health education about the disease is the reason for their misjudgment and ignorance. A study examining the attribution of alcohol consumption claimed that men with higher education levels had lower alcohol consumption and prevalence (He et al., 2022). In a study by Adelaide et al. on educational information, it was stated that health education dissemination through social media or campaigns could increase awareness of the dangers of alcohol and that patients exposed to health information would attempt to consult the internet or medical personnel for more medical education information related to disease risk and health education (Balenger et al., 2023). This reinforces that health education can stimulate patients to learn about the disease, relieve their doubts, and enhance their self-awareness. Some of the older patients in the study believed that their adequate life and social experience was sufficient to cope with the negative effects of the disease, and that blind confidence, a sense of chance, and lack of knowledge were the reasons for their poor adherence and disorganized life management. The younger patients in the study did not have problems with ignorance about the disease. Their education level was generally higher, reinforcing the importance of knowledge acquisition and health education.

Some patients in the study were worried and fearful about disease recurrence or cancer because they felt they could not accurately predict disease progression. Some discomforting symptoms from the disease interfered with their correct judgment and affected their cognitive abilities. An anatomical study discussed the interaction between emotion and cognition, first explaining the influence of brain structure on emotion. They claimed that the extensive connectivity of brain structures amplifies the potential for cognitive and emotional interactions and later elaborated on functionality, proposing the existence of a dual competing architecture for emotion processing, one that views emotion and motivation as combined with perception and cognition, and that these effectively combined interactions allow people to incorporate perceptual values into behavioral patterns effectively (Pessoa, 2010). This is a good explanation at the anatomical level of the tangled and erratic emotions of the patients when confronted with illness, which affects their normal cognitive abilities, and the misperceptions that make them delusional about the disease, cautious and sensitive and emotionally tense in their lives.

Positive long-term encouragement and care from family are essential in the recovery process. Research reveals that having the care and support of family members in sobriety and life management often makes all the difference (Singh et al., 2022). According to a study conducted by Bailey et al. (2017), it has been found that family caregiver involvement plays a crucial role in preventing and managing the risk of chronic diseases, particularly in terms of reducing alcohol consumption among caregivers. The study suggests that family members have a pivotal role in supporting individuals to quit drinking and aiding in their recovery from the disease. However, the realities of life often impose burdens that tend to prioritize the caregiver’s own well-being, leaving them with little choice but to focus less on caring for the patient. Insufficient knowledge about medical services and disease management among individual family members can lead to excessive assistance and overprotectiveness during the management process. Such behavior is undesirable as it may hinder the patient’s progress. While family support is crucial, it is important to strike a balance and accurately gauge the level of care provided, avoiding excessive indulgence. True assistance lies in maintaining an appropriate level of support without crossing the line into overprotectiveness.

Self-motivation and self-determination theories that emerge in sobriety are important reasons why patients experience behavioral changes and adapt to life; motivation is expressed through an individual’s readiness for change, and higher levels of autonomous motivation imply the greatest likelihood of being prepared for sobriety and are likely to portend success (Kushnir et al., 2016). Self-determination theory is an approach to understanding motivation with continuity, which meets the psychological needs of autonomy, relevance, and competence. It transitions from extrinsic to intrinsic motivation, where the patient’s inherent interest, enjoyment, and satisfaction is self-motivated (Walji et al., 2021). In this study, patients experienced transformations in their socialization style and heightened behavioral awareness as a result of positive abstinence and self-referential motivation. Their aim was to overcome the exhaustion associated with their challenging circumstances. The self-determination theory provided them with a positive motivation to abstain, ultimately leading to successful cessation of the habit and overall life improvement. Hence, it can be inferred that adopting a proactive attitude from the outset may serve as a significant stepping stone towards achieving their goals.

Our study had some limitations. Only men with AP and no women were involved in this study, probably because of the small group of women with AP due to alcohol abuse in China. There may also be a risk of recall bias, as in other studies; for example, patients may not remember the specifics of the onset of the disease.

In conclusion, each patient was actively aware of the need to quit drinking and maintain good lifestyle habits. However, due to life’s compulsions, lack of will, or the influence of stigma, they failed to quit drinking, and their disease relapsed or worsened. We provided guidance to help patients correct their roles and improve their self-efficacy. We provided timely knowledge of disease care and life management, psychological guidance, and correct thinking and cognitive guidance to stimulate motivation for change and overcome negative emotions. A positive concept of recovery can inspire patients to perform coping acceptance measures, adopt a new sense of self, and facilitate a reduction in drinking behavior. In addition, we believe it is important to provide individualized management plans that fully integrate the patient’s condition and family relationships. Our findings provide experiential and cognitive insights into the abstinence experience and life management of men with AP, which captures the details of patients’ lives and health pathways for home management and provides a reference for further research to assess aspects of home life management and quality of life after healing in patients with AP.

## Data availability statement

The raw data supporting the conclusions of this article will be made available by the authors, without undue reservation.

## Ethics statement

The studies involving human participants were reviewed and approved by Medical Ethics Committee of Binzhou Medical University. The patients/participants provided their written informed consent to participate in this study.

## Author contributions

RC, QW, and DW performed most of the writing. XYL, HW, JG, and XHL performed data acquisition, writing, and prepared the tables. RC designed the outline and coordinated the writing of the paper. RC and DW contributed to the conception of the study. All authors contributed to the article and approved the submitted version.

## Funding

This work was supported by Natural Science Foundation of Shandong Province (ZR2022MH117), Binzhou Medical University School of Nursing Postgraduate Scientific Research Innovation Support Program (2023-171).

## Conflict of interest

XHL is employed by Shandong Vheng Data Technology Co., Ltd.

The remaining authors declare that the research was conducted in the absence of any commercial or financial relationships that could be construed as a potential conflict of interest.

## Publisher’s note

All claims expressed in this article are solely those of the authors and do not necessarily represent those of their affiliated organizations, or those of the publisher, the editors and the reviewers. Any product that may be evaluated in this article, or claim that may be made by its manufacturer, is not guaranteed or endorsed by the publisher.

## Abbreviations

AP: Alcoholic pancreatitis
ACP: alcoholic chronic pancreatitis
AUDIT-C: Alcohol Use Disorders Identification Test

## References

[ref1] Bailey J. M. Wye P. M. Wiggers J. H. Bartlem K. M. Bowman J. A. (2017). Family carers: a role in addressing chronic disease risk behaviors for people with a mental illness? Prev. Med. Rep. 7, 140–146. doi: 10.1016/j.pmedr.2017.05.014, PMID: 28660122 PMC5480275

[ref2] Balenger A. Scott L. C. Swahn M. H. Aneja R. (2023). Acceptability of primary care counseling and brief educational messages to increase awareness about alcohol and breast Cancer risks among bisexual and lesbian women. Int. J. Environ. Res. Public Health 20:4184. doi: 10.3390/ijerph20054184, PMID: 36901202 PMC10002287

[ref3] Beyer G. Habtezion A. Werner J. Lerch M. M. Mayerle J. (2020). Chronic pancreatitis. Lancet 396, 499–512. doi: 10.1016/S0140-6736(20)31318-0 32798493

[ref4] Brezovec E. Zoričić Z. Glavina T. (2022). Sociability of alcohol consumption and alcoholism in times of COVID-19 crisis. Arch. Psychiatry Res. 58, 99–106. doi: 10.20471/may.2022.58.01.11

[ref5] Broyles L. M. Binswanger I. A. Jenkins J. A. Finnell D. S. Faseru B. Cavaiola A.. (2014). Confronting inadvertent stigma and pejorative language in addiction scholarship: a recognition and response. Subst. Abus. 35, 217–221. doi: 10.1080/08897077.2014.930372, PMID: 24911031 PMC6042508

[ref6] Carol M. Pérez-Guasch M. Solà E. Cervera M. Martínez S. Juanola A.. (2022). Stigmatization is common in patients with non-alcoholic fatty liver disease and correlates with quality of life. PLoS One 17:e0265153. doi: 10.1371/journal.pone.0265153, PMID: 35385510 PMC8986095

[ref7] Chaudhry H. Sohal A. Sharma R. Dukovic D. Lee D. Gamboa A.. (2023). Increased mortality in patients with alcohol-induced pancreatitis during the COVID-19 pandemic. Ann. Gastroenterol. 36, 68–72. doi: 10.20524/aog.2022.0769, PMID: 36593806 PMC9756021

[ref8] Chen L. Zhou X. Tu X. Cheng H. Duan Z. Lu G.. (2022). People's perceptions and experience of managing life after recurrent pancreatitis: a qualitative study in eastern China. Sci. Rep. 12:18749. doi: 10.1038/s41598-022-22287-w, PMID: 36335196 PMC9637212

[ref9] Conwell D. L. Banks P. A. Sandhu B. S. Sherman S. Al-Kaade S. Gardner T. B.. (2017). Validation of demographics, etiology, and risk factors for chronic pancreatitis in the USA: a report of the North American pancreas study (NAPS) group. Dig. Dis. Sci. 62, 2133–2140. doi: 10.1007/s10620-017-4621-z, PMID: 28600657 PMC6040886

[ref10] Cronin P. Begley C. (2013). Living with chronic pancreatitis: a qualitative study. Chronic Illn. 9, 233–247. doi: 10.1177/1742395312465627, PMID: 23129786

[ref11] Fitzsimmons D. Kahl S. Butturini G. van Wyk M. Bornman P. Bassi C.. (2005). Symptoms and quality of life in chronic pancreatitis assessed by structured interview and the EORTC QLQ-C30 and QLQ-PAN26. Am. J. Gastroenterol. 100, 918–926. doi: 10.1111/j.1572-0241.2005.40859.x, PMID: 15784041

[ref12] Gapstur S. M. Jacobs E. J. Deka A. McCullough M. L. Patel A. V. Thun M. J. (2011). Association of alcohol intake with pancreatic cancer mortality in never smokers. Arch. Intern. Med. 171, 444–451. doi: 10.1001/archinternmed.2010.536, PMID: 21403041

[ref13] Graham S. (2022). Self-efficacy and language learning – what it is and what it isn't. Lang. Learn. J. 50, 186–207. doi: 10.1080/09571736.2022.2045679

[ref14] Hayduchok I. Tukhar I. Shapovalov V. (2022). Chronic pancreatitis, comorbid with alcohol addiction: epidemiology, causes, developmental features, symptoms and supportive pharmaceutical therapy. SSP Mod. Pharm. Med. 2, 1–13. doi: 10.53933/sspmpm.v2i2.46

[ref15] He H. Pan L. Ren X. Wang D. Du J. Cui Z.. (2022). The effect of body weight and alcohol consumption on hyperuricemia and their population attributable fractions: a National Health Survey in China. Obes. Facts 15, 216–227. doi: 10.1159/000521163, PMID: 34839297 PMC9021635

[ref16] Iannuzzi J. P. King J. A. Leong J. H. Quan J. Windsor J. W. Tanyingoh D.. (2022). Global incidence of acute pancreatitis is increasing over time: a systematic review and meta-analysis. Gastroenterology 162, 122–134. doi: 10.1053/j.gastro.2021.09.043 34571026

[ref17] Ji C. (2012). Mechanisms of alcohol-induced endoplasmic reticulum stress and organ injuries. Biochem. Res. Int. 2012:216450. doi: 10.1155/2012/216450, PMID: 22110961 PMC3205771

[ref18] Jiang Z. Hu X. Wang Z. Jiang X. (2017). Career decision self-efficacy and life satisfaction in China: an empirical analysis. Soc. Indic. Res. 132, 137–154. doi: 10.1007/s11205-015-1201-5

[ref19] Kushnir V. Godinho A. Hodgins D. C. Hendershot C. S. Cunningham J. A. (2016). Motivation to quit or reduce gambling: associations between self-determination theory and the Transtheoretical model of change. J. Addict. Dis. 35, 58–65. doi: 10.1080/10550887.2016.1107315 26488909

[ref20] Lee C. Y. Lee C. H. Lai H. Y. Chen M. M. (2022). Influence of alcohol provocation on medical professionals in Taiwan: a qualitative study. PLoS One 17:e0264071. doi: 10.1371/journal.pone.0264071 35171965 PMC8849514

[ref21] Lewis A. R. Pihlak R. McNamara M. G. (2018). The importance of quality-of-life management in patients with advanced pancreatic ductal adenocarcinoma. Curr. Probl. Cancer 42, 26–39. doi: 10.1016/j.currproblcancer.2018.01.013, PMID: 29631711

[ref22] Lin R. T. Chen P. L. Yang C. Y. Yeh C. C. Lin C. C. Huang W. H.. (2022). Risk factors related to age at diagnosis of pancreatic cancer: a retrospective cohort pilot study. BMC Gastroenterol. 22:243. doi: 10.1186/s12876-022-02325-7 35568803 PMC9107247

[ref23] Lindgren B. M. Lundman B. Graneheim U. H. (2020). Abstraction and interpretation during the qualitative content analysis process. Int. J. Nurs. Stud. 108:103632. doi: 10.1016/j.ijnurstu.2020.103632, PMID: 32505813

[ref24] Liu J. Zhang B. (2020). The lived experience of inpatients with acute recurrent pancreatitis: a qualitative research study from West China. Gastroenterol. Nurs. 43, 249–257. doi: 10.1097/SGA.0000000000000442, PMID: 32487957

[ref25] Ma S. Yang X. He H. Gao Y. Chen Y. Qin J.. (2022). Psychological experience of inpatients with acute pancreatitis: a qualitative study. BMJ Open 12:e060107. doi: 10.1136/bmjopen-2021-060107, PMID: 35768082 PMC9244672

[ref26] Mérida-López S. Extremera N. Rey L. (2017). Emotion-regulation ability, role stress and teachers' mental health. Occup. Med. (Lond.) 67, 540–545. doi: 10.1093/occmed/kqx125, PMID: 29016826

[ref27] Muniraj T. Aslanian H. R. Farrell J. Jamidar P. A. (2014). Chronic pancreatitis, a comprehensive review and update. Part I: epidemiology, etiology, risk factors, genetics, pathophysiology, and clinical features. Dis. Mon. 60, 530–550. doi: 10.1016/j.disamonth.2014.11.002, PMID: 25510320

[ref28] Nieweglowski K. Corrigan P. W. Tyas T. Tooley A. Dubke R. Lara J.. (2018). Exploring the public stigma of substance use disorder through community-based participatory research. Addict. Res. Theory 26, 323–329. doi: 10.1080/16066359.2017.1409890

[ref29] Nikkola A. Mäkelä K. A. Herzig K. H. Mutt S. J. Prasannan A. Seppänen H.. (2022). Pancreatic secretory trypsin inhibitor (SPINK1) gene mutation in patients with acute alcohol pancreatitis (AAP) compared to healthy controls and heavy alcohol users without pancreatitis. Int. J. Mol. Sci. 23:15726. doi: 10.3390/ijms232415726, PMID: 36555366 PMC9778821

[ref30] Nwosu I. A. Ekpechu J. Njemanze V. C. Ukah J. Eyisi E. Ohuruogu B.. (2022). Self-report on Men's beliefs and perceptions on their alcohol use/misuse in Southeast Nigeria. Am. J. Mens Health 16:15579883221130193. doi: 10.1177/15579883221130193, PMID: 36333918 PMC9638526

[ref31] Oliveira S. E. Carvalho H. Esteves F. (2016). Internalized stigma and quality of life domains among people with mental illness: the mediating role of self-esteem. J. Ment. Health 25, 55–61. doi: 10.3109/09638237.2015.1124387, PMID: 26828897

[ref32] Pessoa L. (2010). Emergent processes in cognitive-emotional interactions. Dialogues Clin. Neurosci. 12, 433–448. doi: 10.31887/DCNS.2010.12.4/lpessoa 21319489 PMC3117594

[ref33] Roberts S. E. Morrison-Rees S. John A. Williams J. G. Brown T. H. Samuel D. G. (2017). The incidence and aetiology of acute pancreatitis across Europe. Pancreatology 17, 155–165. doi: 10.1016/j.pan.2017.01.005, PMID: 28159463

[ref34] Shenton A. K. (2004). Strategies for ensuring trustworthiness in qualitative research projects. Educ. Inf. 22, 63–75. doi: 10.3233/EFI-2004-22201

[ref35] Shrestha B. Dunn L. (2020). The declaration of Helsinki on medical research involving human subjects: a review of seventh revision. J Nepal Health Res Counc 17, 548–552. doi: 10.33314/jnhrc.v17i4.1042 32001865

[ref36] Singh P. Chakraborty B. Sarkhel S. Ray S. Patra P. S. Das K. (2022). Indian outpatients with idiopathic chronic pancreatitis have catastrophic healthcare expenditure, malnutrition, anxiety/depression and work-impairment. Dig. Dis. Sci. 67, 3612–3622. doi: 10.1007/s10620-021-07255-0, PMID: 34581905

[ref37] Singh A. Thakkar K. Pandit N. (2022). Family support is key to overcome alcohol dependence: a social case study. Med. J. Dr. D.Y. Patil Vidyapeeth 15, 276–278. doi: 10.4103/mjdrdypu.mjdrdypu_170_20

[ref38] Tong A. Sainsbury P. Craig J. (2007). Consolidated criteria for reporting qualitative research (COREQ): a 32-item checklist for interviews and focus groups. Int. J. Qual. Health Care 19, 349–357. doi: 10.1093/intqhc/mzm042, PMID: 17872937

[ref39] Vignato J. Inman M. Patsais M. Conley V. (2022). Computer-assisted qualitative data analysis software, phenomenology, and Colaizzi's method. West. J. Nurs. Res. 44, 1117–1123. doi: 10.1177/01939459211030335 34238074

[ref40] Volkow N. D. Gordon J. A. Koob G. F. (2021). Choosing appropriate language to reduce the stigma around mental illness and substance use disorders. Neuropsychopharmacology 46, 2230–2232. doi: 10.1038/s41386-021-01069-4, PMID: 34276051 PMC8580983

[ref41] Walji A. Romano I. Levitt E. Sousa S. Rush B. MacKillop J.. (2021). Psychometric evaluation of the treatment entry questionnaire to assess extrinsic motivation for inpatient addiction treatment. Drug Alcohol Depend. Rep. 2:100014. doi: 10.1016/j.dadr.2021.100014, PMID: 36845886 PMC9949302

[ref42] Wang W. Jiang W. Shu M. Luo L. Liu A. Pan X.. (2021). Regional variations in distribution, diagnosis and treatment of chronic pancreatitis in mainland China: a systematic review of case articles over 40 years. Available at: https://ssrn.com/abstract=3753376.

[ref43] Wang Y. C. Zou W. B. Tang D. H. Wang L. Hu L. H. Qian Y. Y.. (2023). High clinical and genetic similarity between chronic pancreatitis associated with light-to-moderate alcohol consumption and classical alcoholic chronic pancreatitis. Gastroenterol. Hepatol. Adv. 2, 186–195. doi: 10.1016/j.gastha.2022.09.009 PMC11308850 39132611

[ref44] Xiao A. Y. Tan M. L. Wu L. M. Asrani V. M. Windsor J. A. Yadav D.. (2016). Global incidence and mortality of pancreatic diseases: a systematic review, meta-analysis, and meta-regression of population-based cohort studies. Lancet Gastroenterol. Hepatol. 1, 45–55. doi: 10.1016/S2468-1253(16)30004-8, PMID: 28404111

[ref45] Zwick J. Appleseth H. Arndt S. (2020). Stigma: how it affects the substance use disorder patient. Subst. Abuse Treat. Prev. Policy 15:50. doi: 10.1186/s13011-020-00288-0, PMID: 32718328 PMC7385978

